# Effect of urban tourist satisfaction on urban macroeconomics in China: A spatial panel econometric analysis with a spatial Durbin model

**DOI:** 10.1371/journal.pone.0206342

**Published:** 2018-10-31

**Authors:** Min Zhou, Xiaoqun Liu, Guoan Tang

**Affiliations:** 1 School of Architecture, Hunan University, Changsha, Hunan, P. R. China; 2 Tourism School, Hainan University, Haikou, Hainan, P. R. China; University of Sydney, AUSTRALIA

## Abstract

Tourist satisfaction has always been a crucial research issue in the tourism economy. This paper utilizes the Spatial Durbin Model (SDM) to analyze the impact of urban tourism satisfaction on urban macroeconomics from a macro perspective, using quarterly data on tourist satisfaction in 35 large and medium-sized cities along with major urban macroeconomic variables. This study is quite distinct from previous research that focused on constructing a tourist satisfaction index and analyzing the influence factors of tourism satisfaction from the perspective of the micro-level internal composition of tourism. The empirical results show: Firstly, in respect of the impact of urban tourists' satisfaction on the GDP income of cities, the SDM and the SDM with a lagged first-order dependent variable (SDM_dlag) show that the short-term and long-term indirect effects of log-tourist satisfaction are significantly positive, indicating that city satisfaction has a significant positive spatial spillover effect on GDP growth in other cities; Secondly, in respect of the influence of urban tourist satisfaction on the cost of urban life in the SDM, the long-term direct and indirect effects of logarithmic satisfaction are significantly positive, implying, in the long run, that tourist satisfaction has a positive intraregional spillover effect and spatial spillover effect on urban living costs; Finally, the SDM_dlag for the regression of urban tourist satisfaction on the cost of urban daily life shows that the short-run direct and indirect effects of city tourist satisfaction are significantly negative, indicating that tourist satisfaction has intra-regional and spatial spillover effects, and its rise will reduce the cost of living expenses in local and other cities in the short term. Overall, we have further elucidated the role of different levels of urban tourist satisfaction in city macroeconomics from the spatial dimension, thereby enriching the existing research on tourist satisfaction to some certain extent.

## Introduction

The questions of whether high tourist satisfaction may bring about considerable local tourism income and how to facilitate tourist satisfaction have become a main focus within academic circles, both at home and abroad. To explore the factors influencing tourist satisfaction and establish a theoretical and empirical model for evaluating tourist satisfaction is not only conducive to improving the tourism environment, enhancing the attraction of tourism destinations and improving the level of management services, but also facilitates other tourism planning and development projects (Pizam, 1978 [1]; Lee et al., 2007 [2]; Agyeiwaah et al., 2016 [3]; Truong et al., 2017 [4]; Tseng, 2017 [5]; Dong and Yang, 2005 [6]; He, 2011 [7]; Dai et al., 2012 [8]; Luo et al., 2013 [9]; Ma, 2017 [10]).

In the past, the study of tourist satisfaction has mainly unfolded from two aspects in China. Firstly, based on different statistical models, a comprehensive system for evaluating tourist satisfaction has been constructed. For example, Shi and Liu (2009)[11] utilized a pure perception model to study tourist satisfaction. Dai et al. (2012)[8] organized a group of "tourist satisfaction indexes" to concentrate both on the construction of the tourist satisfaction evaluation system and on empirical research; Chen (2013)[12] applied a revised interpretative phenomenological analysis approach to the study of tourist satisfaction.

Secondly, in view of the different tourist attractions at a particular period of time, these papers probed the more detailed factors affecting tourists' satisfaction and constructed some specific tourist satisfaction indexes of these tourism attractions based on certain specific theories and empirical methods. Tourist attractions include natural scenic spots, tourist destinations, ancient towns, theme parks, (historical) cultural and creative tourist destinations, ethnic tourism, (world-wide) heritage sites, urban characteristics, sketching tourists, urban agricultural tourism, rural tourism, self-driving tourism, and intelligent tourism. The tourist satisfaction evaluation models of tourist attraction are as follows: Wang et al.(2006)[13] built a tourist satisfaction index evaluation model of the tourist environment; Zhang (2009) [14] analyzed the construction and evaluation of a tourist satisfaction evaluation index system in ancient towns; Dong et al.(2010)[15] put forward a theme park tourist satisfaction curve; Wang et al.(2011) [16] and Deng(2013)[17] and Luo et al.(2016) [18] constructed an evaluation model of the tourist satisfaction index of cultural and creative tourist destinations; and a measurement model of tourist satisfaction in world heritage sites was provided by He et al.(2013) [19] and He (2014) [20]. On the other hand, the studies of the factors influencing tourist satisfaction with tourist attractions are as follows: the effect of tourists’ host-guest contact preference on perceived destination image and tourist satisfaction (Zhang and Lu, 2010[21]); Analysis of tourist satisfaction of China’s historical and cultural Cities (Cheng and Sun, 2012[22]; Zhang et al., 2014[23]); City features influence the satisfaction of Urban Visitors (Luo et al., 2013[9]); Research on tourist satisfaction in Smart tourism scenic (Su, 2016[24]); Satisfaction Evaluation of Tourist and Influence Factors Analysis in Rural Tourism (Zhou et al., 2016[25]).

Overseas researchers, such as Pizam (1978)[1] and Beard (1980)[26], defined tourist satisfaction through comparison between tourist satisfaction and practical experience in an early age. Baker (2000)[27] and Lee et al. (2007)[2] explored the important relationship between perceived quality, tourist satisfaction, and tourist behavior. Other scholars have concentrated more on environmental problems, sustainability, business tourism and shopping tourism of urban tourism (Hinch,1996 [28]; Jim, 2000 [29]; QU et al.,2000 [30]). However, the amount of research on urban tourism and tourists' satisfaction is relatively small. Bramwell (1998)[31] focused on user satisfaction and product development of international urban tourism; Agyeiwaah et al. (2016)[3] found that there was a significant distinction in tourist satisfaction between the two different sectors of tourist attractions and hotels. Truong et al. (2017)[4] proposed a new concept of uniqueness of tourist destination and explored its positive impact on tourist satisfaction. Tseng (2017)[5] investigated the factors affecting tourism e-customer satisfaction from the perspective of online tourists. Jensen (2017)[32] found that on-site factors make a great difference to visitors’ satisfaction at managed tourist attractions in Northern Norway.

It seems that most of the above domestic and foreign studies on tourist satisfaction have taken the advantage of statistical empirical methods, in which tourism satisfaction is the explanatory variable, and the independent variables are factors influencing tourist satisfaction that are closely related to specific tourism destination attractions. Even so, the modern financial econometric methods shed new light on intriguing more accurate tourism related studies (Lesage and Pace., 2009[33]; Elhorst., 2013[34]; Wen et al., 2014[35]; Huang et al., 2016[36]; Hu et al., 2017[37]; Chao et al., 2017[38]; Fievet et al., 2018[39]; Gong and Lin., 2018[40,41]). This paper’s potential innovation is to analyze the effect of urban tourist satisfaction on the urban macro-economy from the perspective of the spatial dimension, that is to say, using the spatial panel Durbin econometric regression model and taking urban tourist satisfaction as the key independent variable. This is quite distinct from previous studies that have focused on constructing a tourist satisfaction index to investigate the factors influencing tourism satisfaction from the micro-level internal composition of tourism. Moreover, based on relatively high frequency quarterly data from China, we provide empirical evidence from an emerging market complementary to the existing literature on the topic that is predominantly concerned with the U.S. and the European tourism industry markets. This study also probes both the intra-regional spillover effect and the spatial spillover effect of urban tourist satisfaction on both urban GDP income and urban living standards, and it might sheds new light on tourist satisfaction dynamics and facilitate greater understanding of the Tourism industry market rules. Such research is rare.

The reminder of the paper is organized as follows. Section 2 describes the theoretical mechanism and methods, and specifies the spatial econometric model setting. Empirical analysis and results are presented in Section 3, and we conclude and recommend in Section 4.

## Theoriess and methods

### Theoretical mechanism

#### Spatial spillover of urban tourists' satisfaction to urban economic GDP income

In this paper, the urban macro-economy mainly includes urban GDP, consumer price index (CPI), and fixed assets’ investment. Before analyzing the spatial spillover mechanism of urban tourists’ satisfaction on urban economic GDP income, we must first figure out how urban tourists' satisfaction directly affects intra-regional GDP income, as the spatial spillover effect is the extension of the direct effect to a certain extent. Urban tourist satisfaction is one of the significant indicators for measuring the development of urban tourism, and urban characteristics are an essential part of urban tourism (Luo et al., 2013)[9]. A higher degree of tourism satisfaction lays a solid foundation for improving the brand image of urban tourism, building a good reputation for urban tourism, enhancing the loyalty of urban tourists, and promoting revisits by urban tourists, which can both increase urban tourism income, facilitate urban development, and contribute significantly to urban GDP income.

Krugman (1991)[42] argued that spillover effects are never contained within the initial spillover site by either geographical or administrative boundaries. In general, urban tourists' satisfaction may produce a spatial spillover effect through the following two channels: (1) A pure knowledge technology spillover effect—to gain knowledge of tourists’ experiences of urban tourism attractions, urban tourism authorities will travel to cities with similar attractions that have produced a high degree of tourist satisfaction and emulate them; (2) The traffic overflow effect—the convenience of transportation is a crucial factor for tourists’ travel frequency. Marked improvement in the convenience of traffic conditions between adjacent cities will enable the indirect spread of tourist satisfaction from cities with higher tourist satisfaction to neighboring cities.

#### Spatial spillover of urban tourists' satisfaction to the cost of urban life

Similar to the analysis of the spatial spillover mechanism of urban tourists' satisfaction to urban economic GDP income, the enhancement of urban tourists' satisfaction will, firstly, directly affect the cost of living in the city. From the perspective of the construction of the city tourists' satisfaction index, the higher the city tourists' satisfaction, the more reasonable the cost of living should be. Fair city CPI and housing construction enable tourists to enjoy their travelling experience at a reasonable price. Secondly, once highly-satisfied tourists attract more tourists, according to the principle of economic supply and demand, this will inevitably cause a more lasting high cost of living in the city.

As for the spatial spillover effect, including traffic spillover effect and trade spillover effect, of urban tourists' satisfaction on urban living costs, the common point of the two effects is that the improvement of the transportation network and the prevalence of the internet economy makes trade in commodities between the city and the surrounding cities, or even more distant cities, much easier, consequently, the cross-market arbitrage does not exist any longer. Thirdly, the high cost of living of the city triggered by the urban tourist satisfaction will be transmitted to other cities because of the close connections of the cities in terms of economy, trade, culture, politics, and so on.

### Spatial econometric model setting

#### Benchmark model settings and descriptions

The first step of studying the existence of the spatial spillover effect is correctly setting the spatial econometric model. Three kinds of spatial panel models are mainly adopted in empirical research: the spatial lag model, the spatial error model, and the SDM. Based on the specific purpose and theoretical mechanism of the study, this paper attempts to explore the spatial impact of tourist satisfaction on urban macro-economy with the SDM. Firstly, the SDM for tourists' satisfaction to urban economic GDP income is as follows:

$$\ln GDP_{it}=\rho W\ln GDP_{it}+\beta \ln Satisfy_{it}+\theta W\ln Satisfy_{it}+\gamma \ln Invest_{it}+\eta CPI_{it}+\mu_{i}+\delta_{t}+\varepsilon_{it}$$ (1)

$$\varepsilon_{it}∼N(0,\sigma^{2})$$

Where subscript *i* denotes a city, and *t* denotes a quarter; ln*GDP*_*it*_ is the dependent variable, and W is the exogenous space weight matrix; *W*ln*GDP*_*it*_ specifies the weight matrix for the spatial-autoregressive term, and *ρ* is the spatial correlation coefficient indicating the degree of influence between adjacent variables; *W*ln*Satisfy*_*it*_ specifies the weight matrix for the spatially lagged regressor, and *θ* is its spatial coefficient; ln*Invest*_*it*_ and *CPI*_*it*_ are control variables; *μ*_*i*_ and *δ*_*t*_ represent the city individual effect and time fixed effect, respectively; and *ε*_*it*_ is random error term. Given that a distribution function, Anselin (1988)[43] pointed out that a consistent unbiased estimate can be obtained by using maximum likelihood estimation, and the spatial panel maximum likelihood estimation is used in this paper. In fact, the ‘endogeneity problem’ in this case has to do with simultaneous equations, and the formula (1) actually can be interpreted as a ‘typical’ equation of a simultaneous equation model with as many equations as there are cities (i = 1,2,..,n).

In addition, we would apply the Hausman test to decide it is the spatial econometric model with fixed effect or with the random effect model will be employed.

Meanwhile, the SDM of tourist satisfaction to urban economic cost of living is as follows:

$$\ln\;\mathrm{Cos}t_{it}=\rho W\ln\;\mathrm{Cos}t_{it}+\beta \ln Satisfy_{it}+\theta W\ln Satisfy_{it}+\gamma \ln Invest_{it}+\phi \ln GDP+\mu_{i}+\delta_{t}+\varepsilon_{it}$$ (2)

Where, lnCos*t*_*it*_ includes ln*CPI*_*it*_, lnPr*ice*_*it*_, and ln*House*_*it*_; *W*ln*Invest*_*it*_ and *W*ln*GDP*_*it*_ will be added in the specific empirical test.

Finally, the SDM_dlag just adds the lag one-stage dependent variables as explanatory variables into Eqs (1) and (2).

#### Measurement of direct and indirect effects

In a spatial setting, the effect of an explanatory variable change in a particular unit affects not only that unit but also its neighbors. By using the concept of partial differential method in mathematics, Lesage and Pace (2009)[33] propose to measure the direct and indirect effects between adjacent regions. Furthermore, Elhorst (2013)[34] applies this approach to the direct and indirect effects measurement in SDM, taking into account the spatial correlation information of the dependent variables and the independent variables.

According to these researchers’ ideas, the existence of an intra-regional spillover effect is not related to the estimated coefficient of explanatory variables (*β*), but we should pay attention to whether the direct effect of the estimation of explanatory variables is significant. Also, to test for the existence of a spatial spillover effect, we should concentrate on whether the indirect effect of explanatory variables is significant, and not either the spatial autocorrelation coefficient (*ρ*) or the spatial lag coefficient of explanatory variables (*θ*). Assume that the general matrix expression of the SDM model is as shown in formula (3):

$$Y_{it}=\rho WY_{it}+\beta X_{it}+\theta WX_{it}+\mu_{i}+\delta_{t}+\varepsilon_{it}$$ (3)

Where Y_*it*_ is the explained vector, and X_*it*_ represents an explanatory variable vector. ρ is the spatial autocorrelation coefficient, W is the exogenous space weight matrix, and ρW represents ρ multiple W, the use of ρW is calculating the direct and indirect effect, which possesses no economic meaning; however, while Wy_*it*_ also signifies W multiple y_*it*_, it has a true economic meaning, that is, Wy_*it*_ is a new explanatory variable.

It is modified as shown in formula (4):

$$Y_{it}={\left(I-\rho W\right)}^{-1}\left(\beta X_{it}+\theta WX_{it}\right)+{\left(I-\rho W\right)}^{-1}\mu_{i}+{\left(I-\rho W\right)}^{-1}\delta_{t}+{\left(I-\rho W\right)}^{-1}\varepsilon_{it}$$ (4)

Where the interpretations of ρ and θ are the same as Eq (3), and in the process of the model construction, if θ = 0, then, the SDM degrades into the SLM, and if θ+ρβ = 0, it degrades into SEM, otherwise, it still a SDM.

Taking a partial derivative of formula (4) to the k-th explanatory variable, a partial differential matrix is obtained, as shown in formula (4):

$$\left[\frac{\partial Y}{\partial X_{it}}\times \frac{\partial Y}{\partial X_{Nt}}\right]=\left[\begin{array}{c} \frac{\partial Y_{1}}{\partial X_{1t}}\times \frac{\partial Y_{1}}{\partial X_{Nt}}\\ \vdots \\ \frac{\partial Y_{N}}{\partial X_{1t}}\times \frac{\partial Y_{N}}{\partial X_{Nt}} \end{array}\right]={\left(I-\rho W\right)}^{-1}\left[\begin{array}{c} \begin{array}{cccc} \beta_{t} & w_{12}\delta_{t} & \cdots & w_{1N}\delta_{t} \end{array}\\ \begin{array}{cccc} w_{21}\delta_{t} & \beta_{t} & \cdots & w_{2N}\delta_{t} \end{array}\\ \begin{array}{cccc} \vdots & \vdots & \ddots & \vdots \end{array}\\ \begin{array}{cccc} w_{N1}\delta_{t} & w_{N2}\delta_{t} & \cdots & \beta_{t} \end{array} \end{array}\right]$$ (5)

Where the average value of the elements on the main diagonal of the matrix is defined as the direct influence of the *k*-*th* explanatory variable on the explained variable in the region; the average value of elements other than diagonal lines is defined as the indirect effect of the explanatory variable affected by other regions.

#### Setting of spatial weight matrix *W*

Regional agglomeration characteristics, namely spatial autocorrelation, indicate whether there are significant spatial spillover and mutual imitation effects in the variables. We calculated Moran's I index to test for the existence of the spatial correlation (Lesage and Pace., 2009[33]). Moran's I index is defined as follows:

Where *X* is variable, including both the explained and explanatory variables, and *W* represents an adjacent order matrix with n×n spaces, the key issue becomes how to choose a reasonable *W*. Either the contiguity based spatial weight matrix (*W*1) or a distance based spatial weights (*W*2) are widely used. In this paper, we use the former, and we find that the empirical results do not change when the alternative is applied. *W*1 is expressed as the following:

$$W_{ij}=\{\begin{array}{c} 1,regions\;i\;and\;j\;are\;neighboring\;regions\;\;\;\;\\ 0,regions\;i\;and\;j\;are\;not\;neighboring\;regions \end{array}$$

In this paper, Moran's I index of all explanatory variables and dependent variables is calculated, with the aim of judging whether the spatially lagged term of the variables should be taken as an additional explanatory variable. At the same time, a statistical test for the significance of Moran's I index needs to be conducted; this is usually achieved by a Z test of normal distribution.

### Specific variables

#### Dependent variable

The explained variables in this paper are the two main macroeconomic variables: the city GDP, representing the city's economic income, and the price index representing the city's cost of living, using the city CPI, the city commodity sales price index (Price), and the city housing sales price index (Housing).

#### Key explanatory variable

This paper focuses on the spatial impact of urban tourist satisfaction on urban economic income and urban cost of living; thus, the core explanatory variable is urban tourist satisfaction and its spatially lagged term. The tourist satisfaction index of 35 large and medium-sized cities primarily originates from the CSMAR database.

#### Control variables

According to Solow's growth model of macroeconomics, one of the most important factors in studying the influence of urban economic income and urban living cost is urban fixed assets’ investment (Solow, 1956[44]). Consequently, this variable is a crucial control variable in this paper, and, because this paper discusses the spatial influence, if the spatially lagged term of urban fixed assets’ investment passes Moran's I index spatial autocorrelation test, then it may also become one of the control variables. Unfortunately, quarterly urban employment data, which is also a key variable in Solow's model, are not available and are not taken into account.

Then, when the urban GDP is a dependent variable, the urban cost of living will be added to the model as a control variable to preliminarily explore the relationship between the urban cost of living and the satisfaction of urban tourists. On the other hand, when the urban cost of living is a dependent variable, the urban GDP will also be introduced into the regression equation as a control variable.

Finally, compared with other price indexes, the urban housing sales price index can not only represent the cost of living but can also be regarded as a rough yet reasonable proxy variable of urban construction investment, which helps greatly for tourism investment.

## Empirical analysis

### Data and descriptive statistics

This paper uses quarterly data on tourist satisfaction in 35 large and medium-sized cities’ and their main macro-economic variables (such as urban GDP, urban CPI, urban housing construction sales price index, and urban fixed assets investment) as sample data; the samples spanned from 2010 to 2015 depending on the availability of the data, primarily originating from the CSMAR database, the City Bureau of Statistics, and the Provincial Bureau of Statistics. To make the data comparable and minimize heteroscedasticity, all the variables were logarithmically transformed. Table 1 reports the mean, the standard deviation, the maximum, and the minimum of the variables. As shown, the mean of Lnsatisfy, Lninvestment, Lngdp and Lncpi are 4.32, 6.48, 7.02 and 4.64, respectively. On the other hand, while the standard deviation of Lnsatisfy and Lncpi are relatively small, the Lninvestment and Lngdp possess a high deviation.

**Table 1:** Descriptive statistics of the variables.

Variable	Obs	Mean	Std. Dev.	Min	Max
Lnsatisfy	735	4.3266	0.0624	4.1573	4.4740
Lninvestment	735	6.4801	0.9940	0.7751	12.2126
Lngdp	735	7.0187	0.8452	0.8014	8.8471
Lncpi	735	4.6356	0.0133	4.6042	4.6840
Lnprice	735	4.6271	0.0164	0.5839	4.6840
Lnhouse	735	4.6382	0.0632	0.4909	5.1047

Lnsatisfy, Lninvestment, LnGDP, LnCPI, Lnprice, and Lnhouse represent logarithmic urban tourist satisfaction, logarithmic urban fixed investment, logarithmic urban GDP, logarithmic urban CPI, logarithmic urban commodity sales price index, and logarithmic urban housing construction sales price index, respectively.

### Empirical results

#### Impact of urban tourist satisfaction on urban GDP

The explanatory variables’ Moran's I indexes are calculated by GeoDa software. The Moran's I index is between (-1,1); its zero value indicates that there is no spatial correlation, while greater than zero and less than zero represent positive and negative spatial correlations, respectively.

The Moran's I indexes of the logarithm of tourist satisfaction, GDP, CPI, and investment of each city in China in 2010 to 2014 are shown in **Tables 2–**5. Table 2 and Table 5 indicates that the Moran's I values of the logarithm of the monthly GDP and investment are above 2 and highly significant, respectively; thus, the two possess positive spatial correlation. In addition, Table 3 shows that most of the logarithms of the monthly urban tourist satisfaction are significantly positive; however, three of them are statistically insignificant, with a negative value. As for the Moran's I value of the logarithm of the monthly CIP, the results are complicated; in some months, they are quite positively significant with a higher value, above 4 and 5, while, in other months, the opposite occurs. Thus, three different empirical models will be used for analysis, according to the Moran' s I value of CPI. Lastly, the Moran's I value of the logarithm of the monthly city commodity sales price index (Price) and the city housing sales price index (Housing) are not statistically significant at all.

**Table 2:** Moran's I of GDP from 2010 to 2015 quarterly.

Quarter	Moran's I	Z statistic	p-value	Quarter	Moran's I	Z statistic	p-value
201003	0.287***	2.553	0.005	201212	0.266***	2.399	0.008
201006	0.312***	2.75	0.003	201303	0.287***	2.546	0.005
201009	0.273***	2.439	0.007	201306	0.3***	2.65	0.004
201012	0.293***	2.61	0.005	201309	0.244**	2.204	0.014
201103	0.292***	2.586	0.005	201312	0.242**	2.198	0.014
201106	0.311***	2.739	0.003	201406	0.245**	2.215	0.013
201109	0.268***	2.401	0.008	201409	0.245**	2.215	0.013
201112	0.264***	2.376	0.009	201412	0.23**	2.101	0.018
201203	0.29***	2.571	0.005	201503	0.292***	2.579	0.005
201206	0.312***	2.748	0.003	201506	0.306***	2.702	0.003
201209	0.254	2.289	0.011				

***, **, and * represent the 1%, 5%, and 10% significance levels, respectively, of the Z statistic Test. The same is true for the following tables.

**Table 3:** Moran's I of tourist satisfaction from 2010 to 2015 quarterly.

Quarter	Moran's I	Z statistic	p-value	Quarter	Moran's I	Z statistic	p-value
201003	0.112	1.128	0.13	201212	0.294***	2.602	0.005
201006	0.203**	1.875	0.03	201303	0.241**	2.161	0.015
201009	0.256**	2.312	0.01	201306	0.316***	2.777	0.003
201012	0.13	1.28	0.1	201309	0.324***	2.915	0.002
201103	0.263***	2.372	0.009	201312	0.061	0.73	0.233
201106	0.208**	1.89	0.029	201406	0.141*	1.37	0.085
201109	-0.048	-0.147	0.441	201409	0.199**	1.861	0.031
201112	-0.123	-0.749	0.227	201412	0.237**	2.237	0.013
201203	-0.037	-0.058	0.477	201503	0.27***	2.463	0.007
201206	0.134*	1.323	0.093	201506	0.115	1.184	0.118
201209	0.178**	1.667	0.048				

**Table 4:** Moran's I of CPI from 2010 to 2015 quarterly.

Quarter	Moran's I	Z statistic	p-value	Quarter	Moran's I	Z statistic	p-value
201003	0.189**	1.763	0.039	201212	0.033	0.504	0.307
201006	0.083	0.906	0.183	201303	0.408***	3.543	0.000
201009	0.107	1.100	0.136	201306	0.474***	4.09	0.000
201012	0.163*	1.553	0.06	201309	0.456***	3.926	0.000
201103	0.152*	1.507	0.066	201312	0.423***	3.645	0.000
201106	-0.098	-0.567	0.285	201406	0.169*	1.611	0.054
201109	-0.132	-0.835	0.202	201409	0.133*	1.314	0.094
201112	-0.076	-0.379	0.352	201412	0.142*	1.383	0.083
201203	-0.074	-0.38	0.352	201503	0.047	0.62	0.268
201206	-0.151	-1.032	0.151	201506	0.111	1.147	0.126
201209	-0.07	-0.332	0.37				

**Table 5:** Moran's I of Investment from 2010 to 2015 quarterly.

Quarter	Moran's I	Z statistic	p-value	Quarter	Moran's I	Z statistic	p-value
201003	0.486***	4.147	0.000	201212	0.25**	2.287	0.011
201006	0.336***	2.94	0.002	201303	0.525***	4.49	0.000
201009	0.334***	2.931	0.002	201306	0.316***	2.782	0.003
201012	0.443***	3.844	0.000	201309	0.315***	2.787	0.003
201103	0.515***	4.383	0.000	201312	0.248**	2.256	0.012
201106	0.394***	3.419	0.000	201406	0.376***	3.272	0.001
201109	0.365***	3.197	0.001	201409	0.326***	2.896	0.002
201112	0.3***	2.672	0.004	201412	0.23**	2.103	0.018
201203	0.522***	4.454	0.000	201503	0.519***	4.435	0.000
201206	0.159**	1.8	0.036	201506	0.331***	2.901	0.002
201209	0.383***	3.347	0.000				

To further investigate the characteristics of the autocorrelation of local tourist satisfaction and GDP and investment, etc., a Moran's I scatterplot was generated to depict local spatial autocorrelation features. The four quadrants of the Moran scatterplot reflect the spatial connection of the region to its neighboring regions. The data points in quadrant I to quadrant 4 represent the HH type, the LH type, the LL type, and the HL type, respectively. The first and third quadrants reflect the positive spatial autocorrelation.

**Fig 1:**
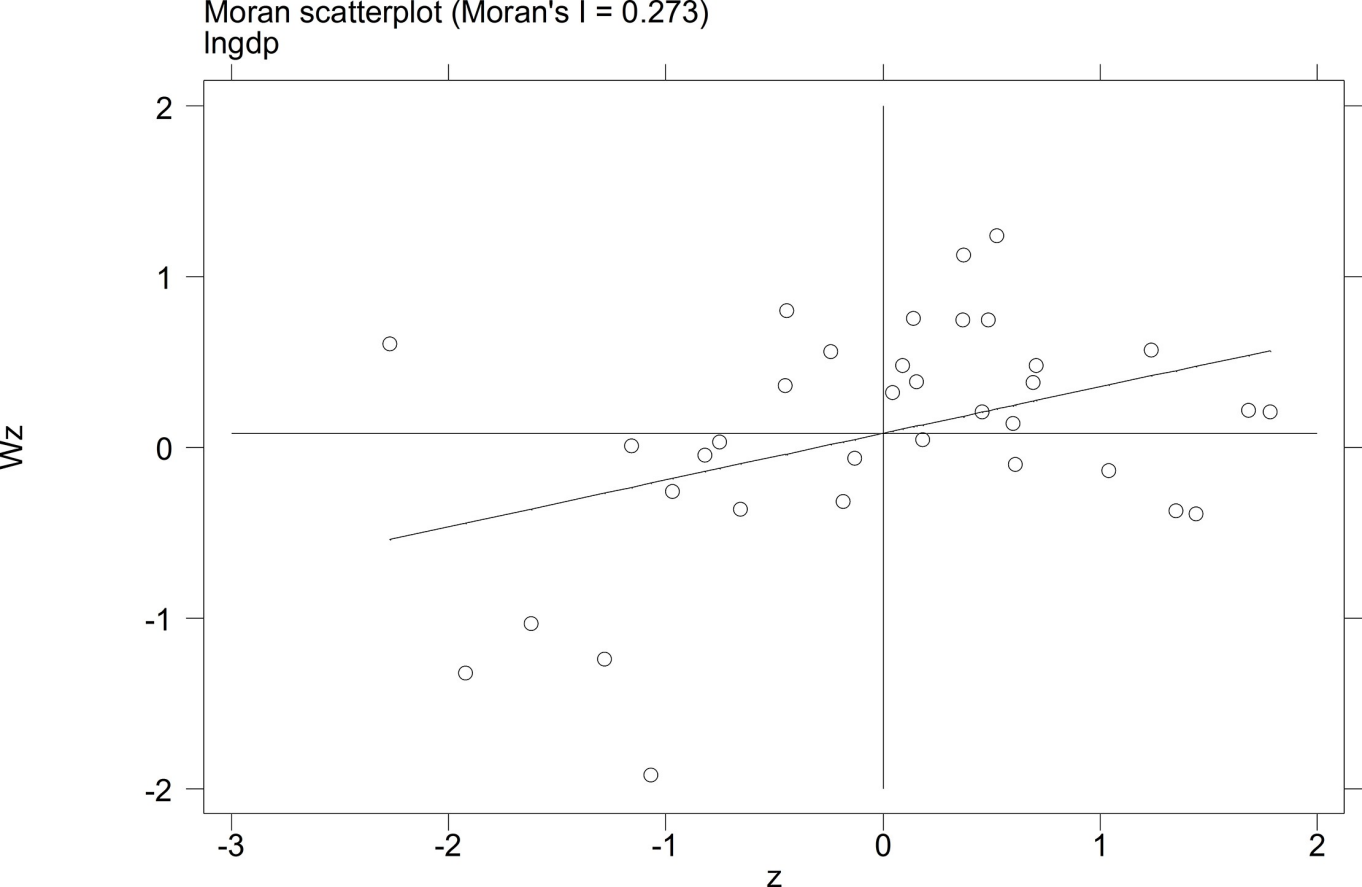
Scatterplot of Moran's I for GDP in Sep. 2010.

**Fig 2:**
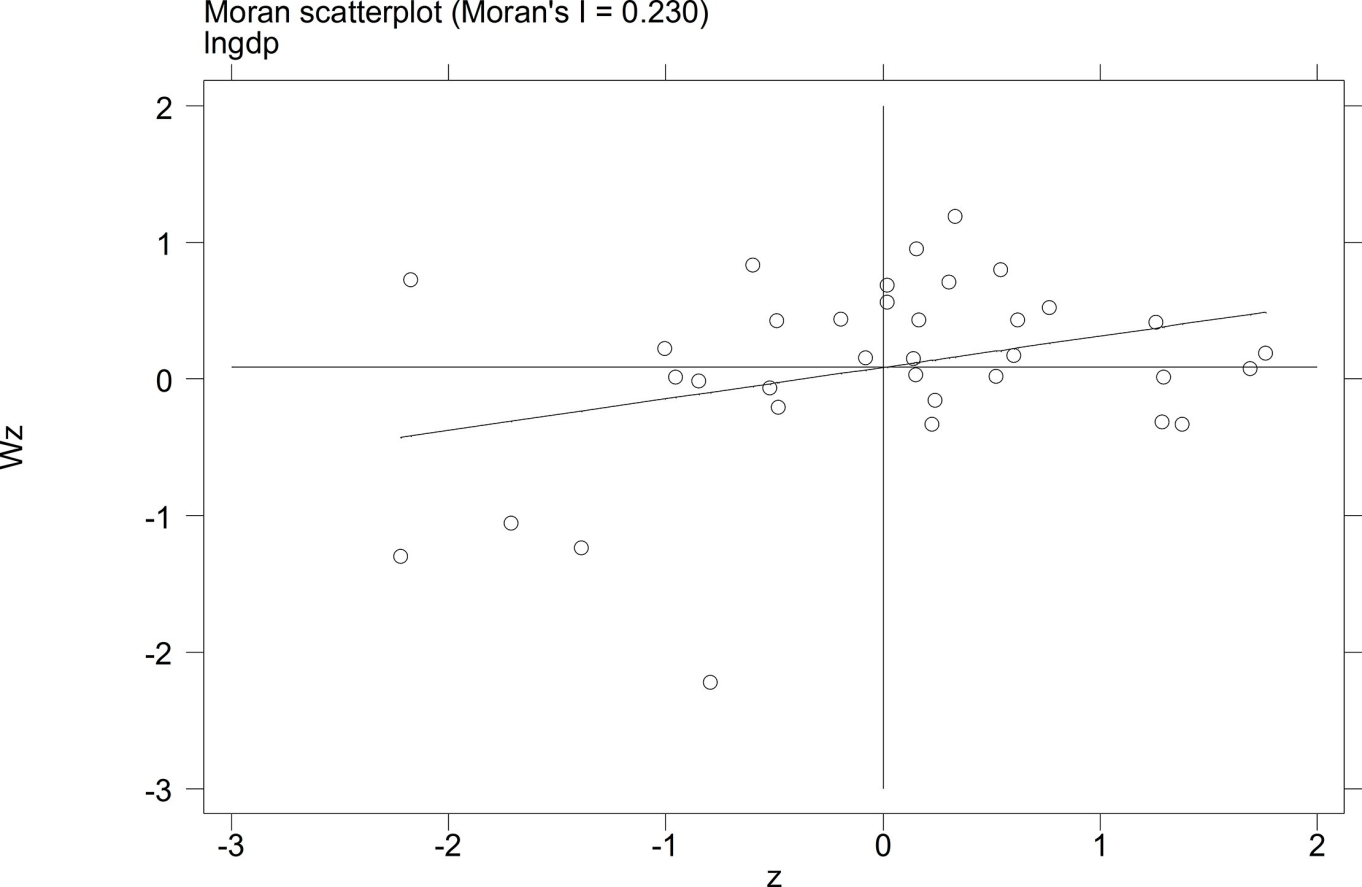
Scatterplot of Moran's I for GDP in Dec. 2014.

**Fig 3:**
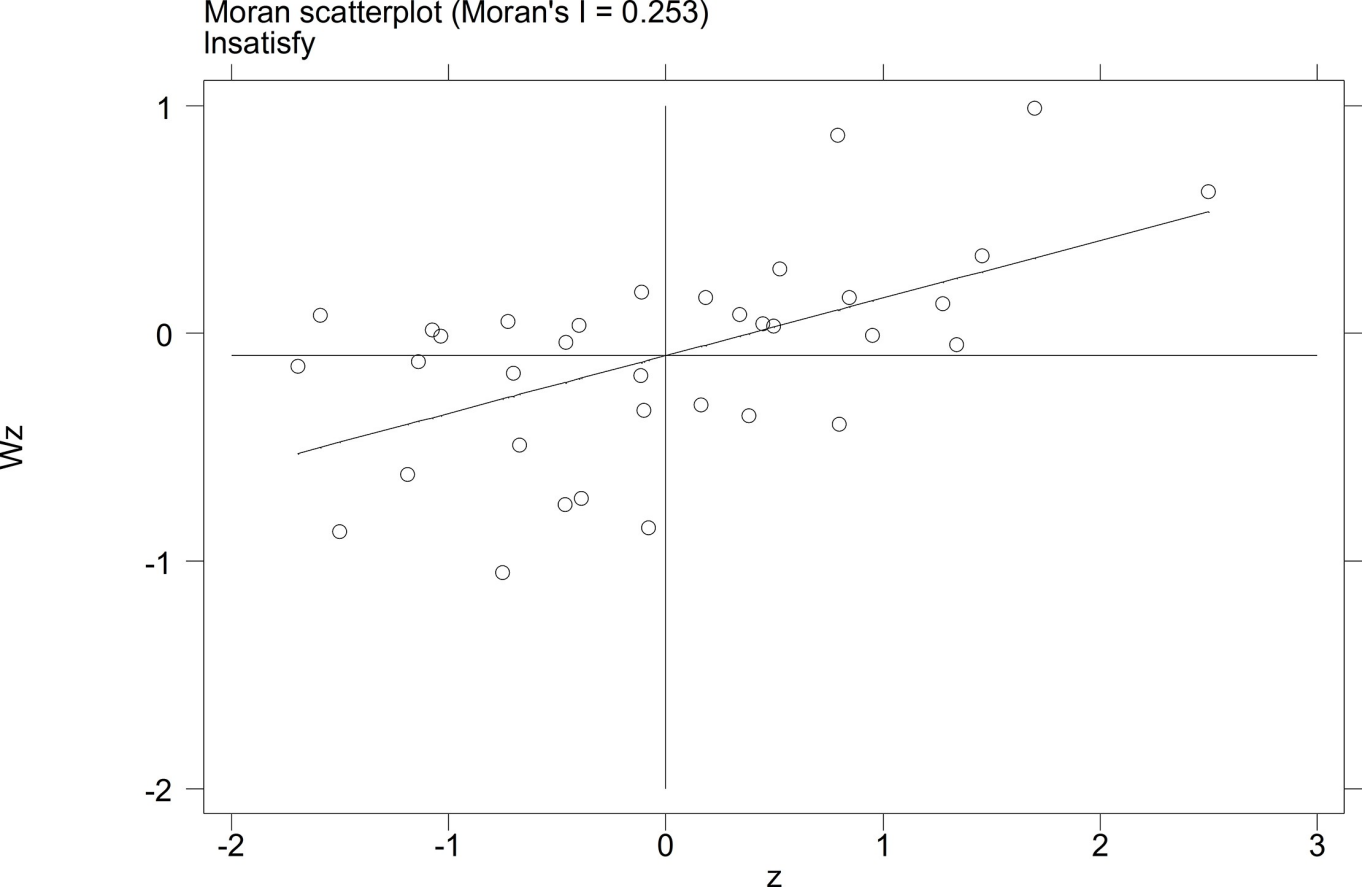
Scatterplot of Moran' I for tourist satisfaction in Sep.2010.

**Fig 4:**
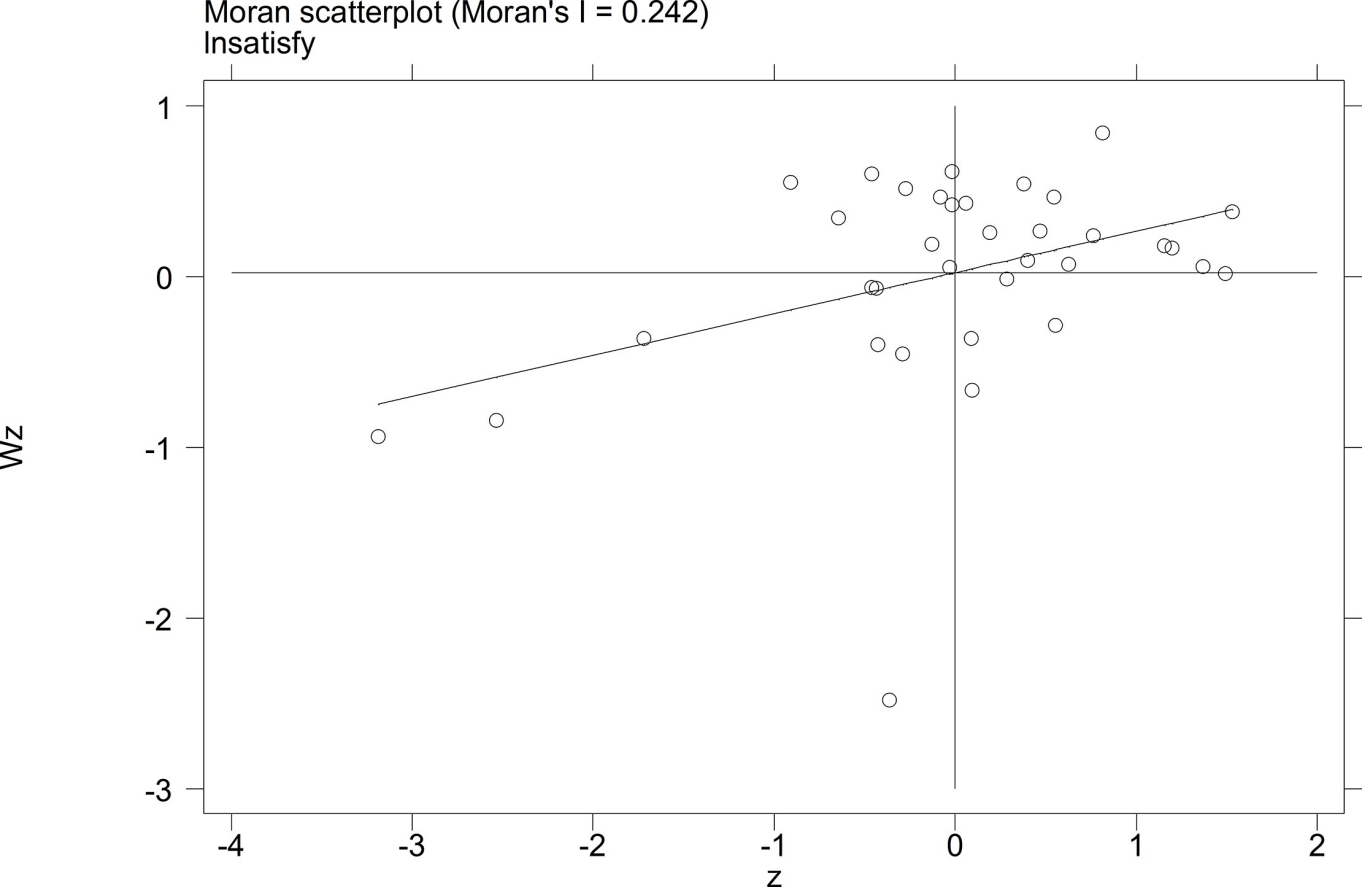
Scatterplot of Moran' I for tourist satisfaction in Dec. 2014.

**Fig 5:**
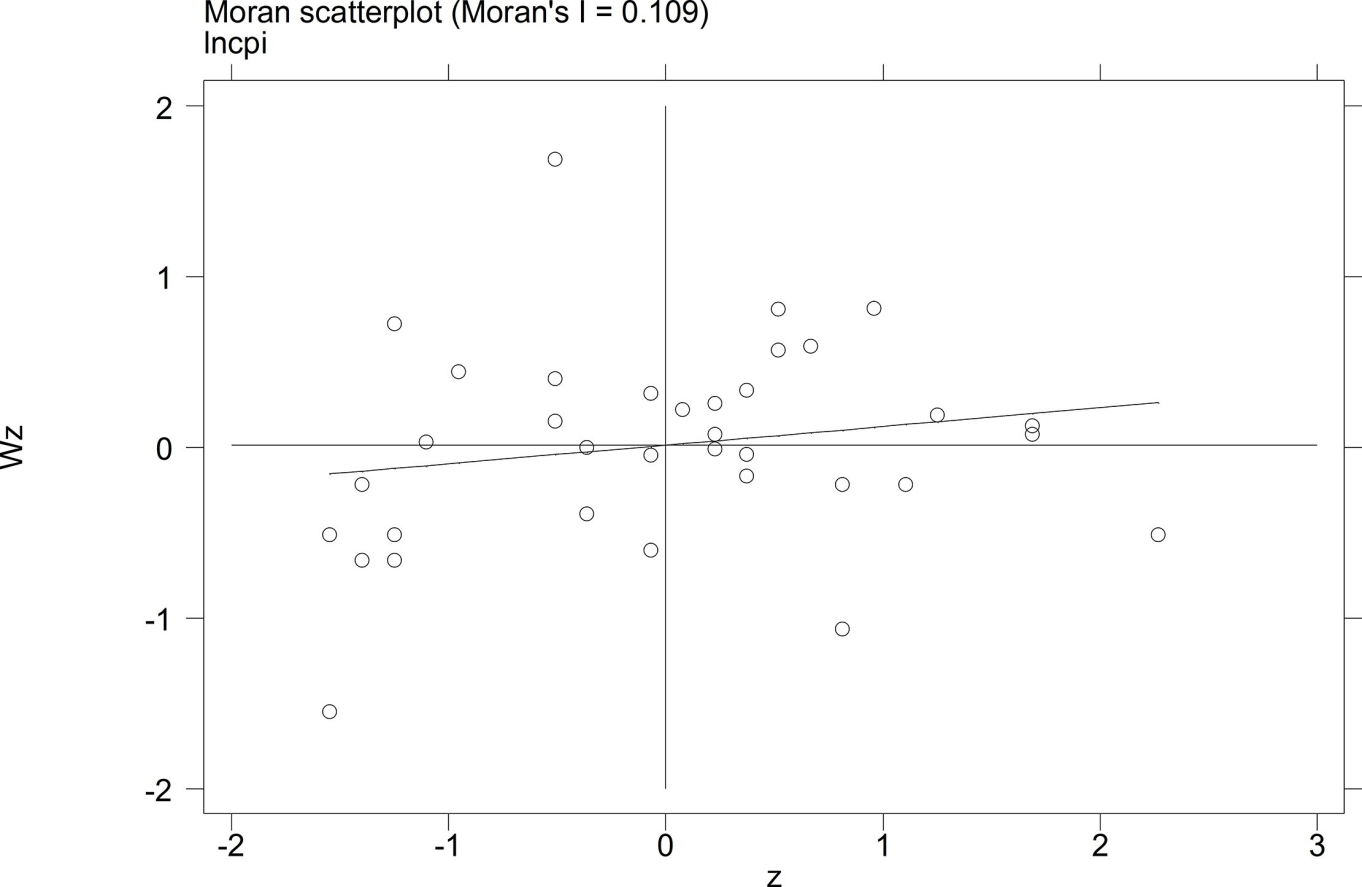
Scatterplot of Moran' I for CPI in Sep.2010.

**Fig 6:**
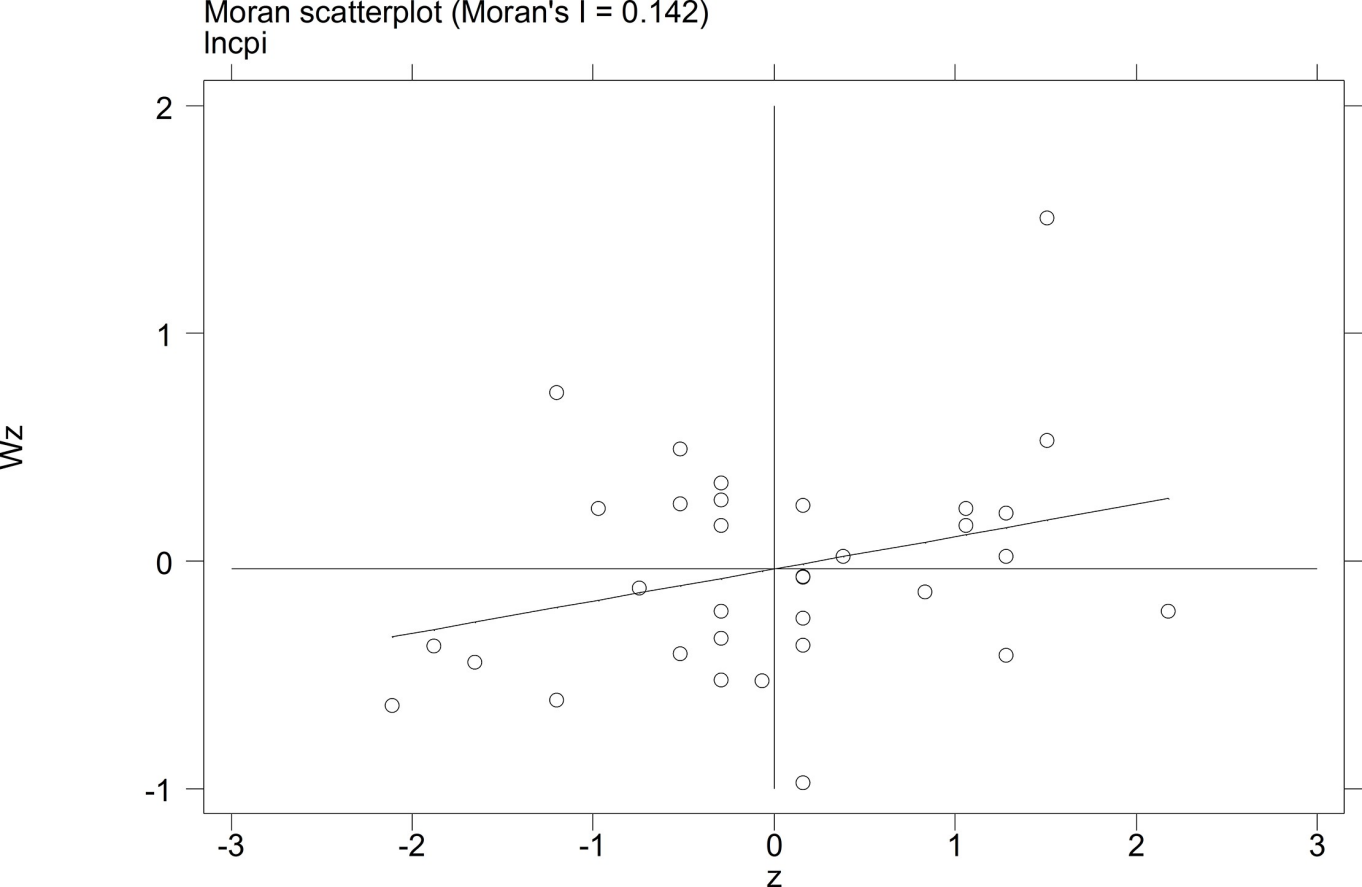
Scatterplot of Moran' I for CPI in Dec. 2014.

**Fig 7:**
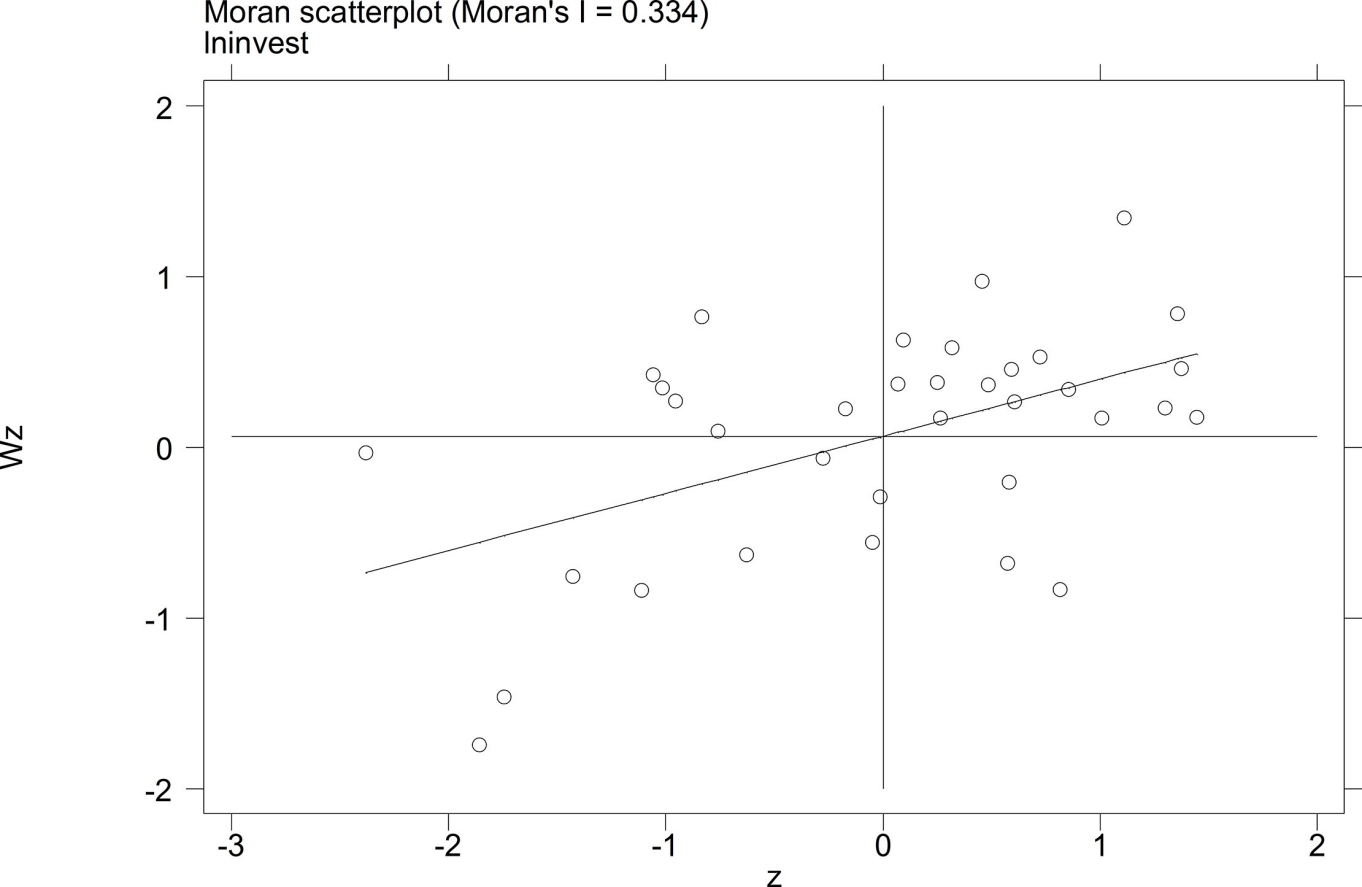
Scatterplot of Moran' I for invest in Sep. 2010.

**Fig 8:**
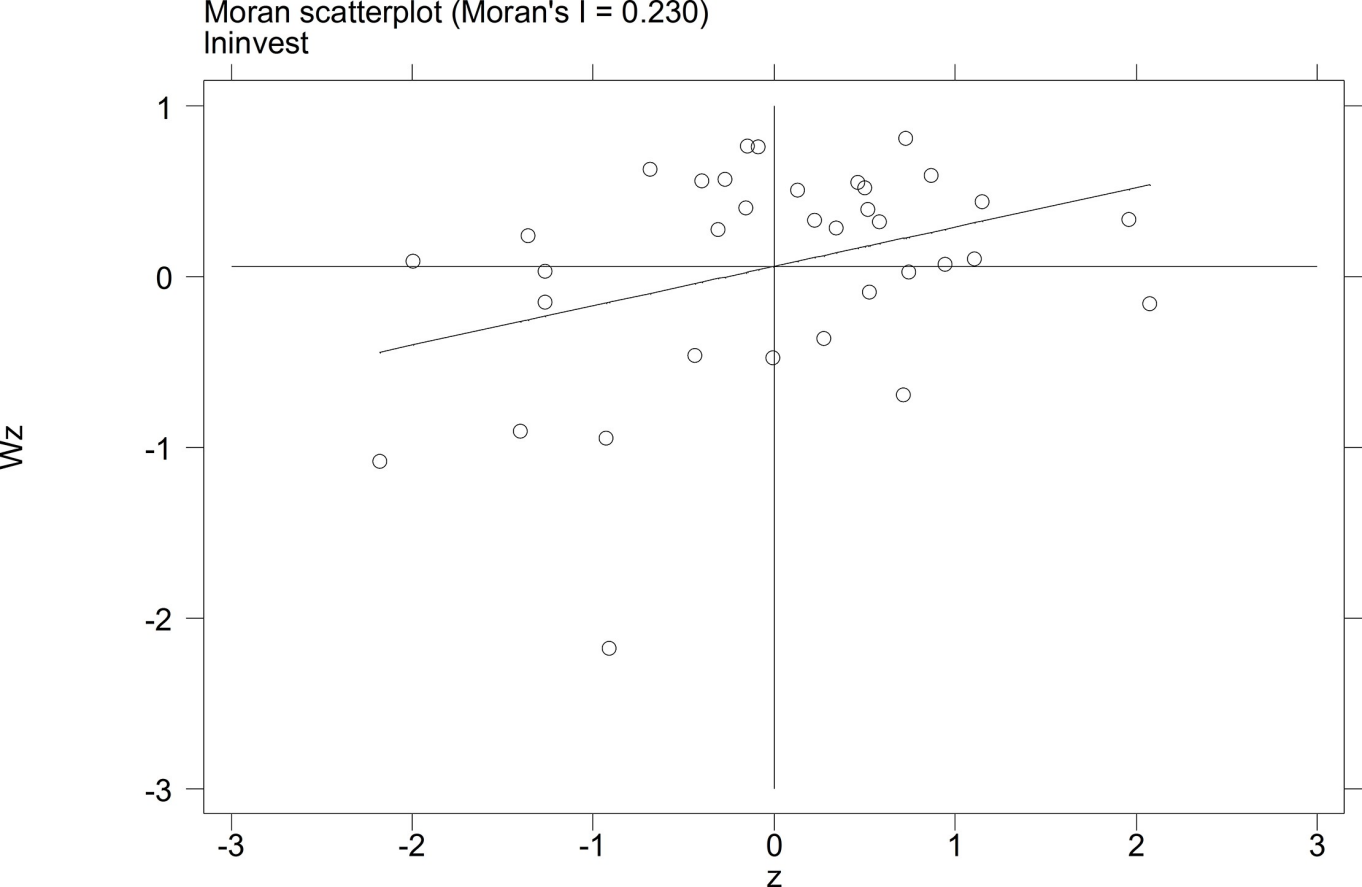
Scatterplot of Moran' I for invest in Dec. 2014.

The Moran scatterplots of the logarithm of the tourist satisfaction, GDP, CPI, and investment of each city in China in 2010 and 2014 are shown in **Figs 1–8**. For most of the 35 cities, the HH and the LL types account for the variables of tourist satisfaction, GDP, and investment. Moreover, as time goes by, an increasing number of cities transfer to the first quadrant (HH type) of each variable, indicating that the development of tourism economy in China's cities has a distinct agglomeration effect; that is, the spatial differentiation characteristics are obvious.

According to the Moran's I value of CPI, three models in **Table 6** are applied for analysis. As the Moran's I value of CPI shows positive spatial autocorrelation only in eight quarters of 2013 and 2014, W* lnCPI will be considered in the Durbin variable of model 1, but not in model 2. Model 3 does not take CPI into account, and its empirical results are compared with those of model 1 and model 2 to preliminarily test the impact of CPI on urban tourists' satisfaction, which helps us probe the impact of urban tourists' satisfaction on the cost of living of urban economy in the following second part.

**Table 6:** SDM regression results for urban tourist satisfaction and urban GDP.

	Model 1		Model 2		Model 3	
Lngdp	Coef.	Z	Coef.	Z	Coef.	Z
**Main**						
Lninvestment	0.0451***	4.67	0.0448***	4.64	0.0446***	4.62
Lnsatisfy	-0.1569	-1.43	-0.1634	-1.49	-0.1668	-1.52
**Lncpi**	**0.2522**	**0.42**	**-0.2594**	**-0.8**		
**Wx**						
Lngdp	0.8978***	11.73	0.9006***	11.77	0.9063***	11.9
Lninvestment	-0.0074	-0.56	-0.0076	-0.58	-0.0085	-0.64
Lnsatisfy	0.2960**	2.23	0.2882**	2.17	0.2593**	2.03
Lncpi	-0.6474	-1.01				
**LR_Direct**						
Lninvestment	0.0455***	4.58	0.0452***	4.56	0.0450	4.54***
Lnsatisfy	-0.1456	-1.39	-0.1518	-1.45	-0.1720	-1.62
Lngdp	-0.0010	-0.06	-0.0009	-0.06	-0.0025	-0.15
**Lncpi**	**0.2284**	**0.39**	**-0.2732**	**-0.87**		
**LR_Indirect**						
Lninvestment	-0.0074	-0.58	-0.0076	-0.6	-0.0093	-0.7
Lnsatisfy	0.2864**	2.16	0.2857**	2.25	0.2635**	2.19
Lngdp	0.8977***	26.43	0.9004***	26.15	0.9106***	27.75
Lncpi	-0.6120	-0.97	-0.0009	-0.03		
**Hausman test**	30.5200***	0.0002	19.98***	0.0056	20.5***	0.0023
**R**^**2**^	0.8443		0.8441		0.8439	
**Log-likelihood**	644.6838		644.173		616.2251	

**Main** are explanatory variables without the Durbin terms; **Wx** represents the weight matrix for the spatially lagged dependent and independent variables; **LR_Direct and LR_Indirect** are the long-run direct effect and indirect effect of the variables; Coef. is the coefficient of the explanatory variable; Z is the critical value of the Z test; and **R**^**2**^ signifies the fitness of the whole model. All these symbols are the same for the following tables.

The empirical regression of Model 1 manifests that the spatially lagged terms of the logarithmic GDP and logarithmic tourist satisfaction are significant, however, the variables’ direct effect and indirect effect matter. Both the logarithmic GDP and the logarithmic tourist satisfaction’s indirect effect are positively and negatively significant; that is, enhancement of urban tourists' satisfaction will also positively affect other cities’ GDP. In other words, urban satisfaction has a significant spatial spillover effect. A city's tourist satisfaction will indirectly promote the GDP of surrounding cities, and cities farther away, through a public praise effect, industrial association, similar urban construction, and other spatial spillover mechanisms (Zhou et al., 2017[45]).

The results of model 2 and model 3 are similar to those of model 1, and the significance of the tourist satisfaction has been facilitated to some extent without adding spatially lagged CPI to the Durbin term. Among the three models, logarithmic investment is the only variable whose direct effect is statistically significant, and the significance decreases when CPI itself and its spatially lagged term are included. This indicates that the impact of fixed assets’ investment, including construction investment, on economic growth is affected by price level. The Hausman test demonstrates that all three models should be SDM with fixed effect. The goodness of fit of the model is higher than 0.8, implying that it is a pretty suitable model from the perspective of the econometrics (Wen et al., 2016[46]).

In measuring the cost of living in the city, there is an urban commodity consumption price index (lnprice) and an urban housing sales price index (lnhouse), in addition to the CPI mentioned above. Therefore, this paper uses “lnprice” and “lnhouse”, together with logarithmic tourist satisfaction and logarithmic fixed assets’ investment, as explanatory variables in models 4 and 5, respectively. The results are depicted in Table 7.

**Table 7:** SDM regression results for urban tourist satisfaction and GDP (continued).

	Model 4			Model 5	
Lngdp	Coef.	z	lngdp	Coef.	z
**Main**			Main		
lninvestment	0.0457***	4.74	lninvestment	0.0451***	4.66
**Lnprice**	-0.6414**	-2.15	**lnhouse**	-0.0935	-1.3
Lnsatisfy	-0.1522	-1.39	lnsatisfy	-0.1791	-1.63
**Wx**			Wx		
Lngdp	0.8784***	11.37	lngdp	0.8941	11.65
lninvestment	-0.0043	-0.32	lninvestment	-0.0070	-0.53
Lnsatisfy	0.3484***	2.6	lnsatisfy	0.2211*	1.69
**LR_Direct**			LR_Direct		
lninvestment	0.0461***	4.66	lninvestment	0.0455***	4.58
Lnprice	-0.6550**	-2.27	lnhouse	-0.0967	-1.39
Lnsatisfy	-0.1405	-1.34	lnsatisfy	-0.1681	-1.6
Lngdp	-0.0010	-0.06	lngdp	-0.0009	-0.06
**LR_Indirect**			LR_Indirect		
lninvestment	-0.0042	-0.33	lninvestment	-0.0069	-0.54
Lnprice	-0.0026	-0.05	lnhouse	-0.0004	-0.04
Lnsatisfy	0.3466***	2.69	lnsatisfy	0.2156*	1.74
Lngdp	0.8778***	23.89	lngdp	0.8938***	25.42
**Hausman test**	26.94***	0.0003		23.6***	0.0013
**R**^**2**^	0.8449			0.8443	
**Log-likelihood**	646.1588			644.6936	

It is worth noting that, because the Moran's I index values of “lnprice” and “lnhouse” are very small, their spatially lagged terms are not considered. The empirical results show that: (1) The Hausman effect indicates that SDM with fixed effect should be introduced, and the goodness of fit of the model is also higher than 0.8; (2) the indirect effect of tourists' satisfaction is also positively significant in the two models, implying that a spatial spillover effect between cities does exist; moreover, tourists' satisfaction is significantly enhanced in model 4, and the coefficient is also larger than before.

#### Impact of urban satisfaction on urban cost of living

The cost of living is measured by logarithmic CPI (lnCPI), the urban commodity consumer price index (lnprice), and the urban residential sales’ price index (lnhouse), respectively. According to the Hausman test, model 6 in Table 8 is the SDM with fixed effect to explore the impact of urban tourist satisfaction on urban CPI, and models 7 and 8 in Table 8 are the SDM with random effect to probe the effect of urban tourist satisfaction on urban CPI and urban housing sales price index, respectively.

**Table 8:** SDM regression results for urban tourist satisfaction and urban cost of living.

	Model 6			Model 7			Model 8	
Lncpi	Coef.	z	lnprice	Coef.	z	lnhouse	Coef.	z
**Main**			**Main**			**Main**		
Lngdp	0.0003	0.12	lngdp	-0.0040***	-3.76	lngdp	-0.0021	-0.51
lninvestment	0.0000	-0.05	lninvestment	0.0005	0.69	lninvestment	0.0002	0.06
Lnsatisfy	-0.0024	-0.35	lnsatisfy	-0.0046	-0.55	lnsatisfy	-0.0393	-1.02
			_cons	1.1352***	12.54	_cons	2.3210***	9.91
**Wx**			**Wx**			**Wx**		
Lngdp	-0.0058**	-2.18	lngdp	-0.0027	-1.65	lngdp	-0.0024	-0.37
lninvestment	0.0011	1.31	lninvestment	0.0006	0.57	lninvestment	-0.0037	-0.78
Lnsatisfy	0.0353***	4.25	lnsatisfy	0.0552	5.44	lnsatisfy	-0.1225**	-2.55
**LR_Direct**			**LR_Direct**			**LR_Direct**		
Lngdp	-0.0018	-0.8	lngdp	-0.0059	-4.44	lngdp	-0.0030	-0.59
lninvestment	0.0004	0.56	lninvestment	0.0008	1.06	lninvestment	-0.0010	-0.27
Lnsatisfy	0.0116*	1.68	lnsatisfy	0.0138	1.72	lnsatisfy	-0.0780**	-2.13
**LR_Indirect**			**LR_Indirect**			**LR_Indirect**		
Lngdp	-0.0209***	-3.44	lngdp	-0.0178***	-3.69	lngdp	-0.0099	-0.57
lninvestment	0.0040	1.64	lninvestment	0.0031	1.2	lninvestment	-0.0091	-0.88
Lnsatisfy	0.1262***	6.05	lnsatisfy	0.1649***	6.98	lnsatisfy	-0.3995***	-4.14
**Hausman test**	33.95***	0	**Hausman test**	10.13	0.1816	**Hausman test**	4.97	0.6637
**R**^**2**^	0.253		**R**^**2**^	0.3580		**R**^**2**^	0.2468	
**Log-likelihood**	615.7		**Log-likelihood**	2396.1		**Log-likelihood**	1244.3	

**SR_Direct and SR_Indirect** are the short-run direct effect and indirect effect of the variables, and these two symbols are the same for the Table 3.5.

In model 6, the direct effect of logarithmic tourist satisfaction is significantly positive, indicating that the effect of enhancement of tourist satisfaction on a city's CPI is positive; i.e., the improvement of tourist satisfaction will lead to a much higher city CPI, which will inevitably give rise to a relatively higher cost of living. Thus tourist, satisfaction has an intra-regional spillover effect on urban CPI. This can be understood from the following logic: when constructing the tourist satisfaction index, CPI is supposed to be one of its influencing factors—the lower the CPI, the greater the marginal impact of tourist satisfaction—making the city attract more tourists. However, in the long run, the greater number of tourists, the greater the consumption, which will bring about a higher CPI eventually. At the same time, the indirect effect of tourist satisfaction is also positively significant, that is, the reinforcement of urban tourist satisfaction will also have a positive effect on CPI in other cities. In other words, urban tourist satisfaction has a prominent spatial spillover effect. The satisfaction of tourists in a certain city will indirectly drive the price rise of neighboring cities and cities farther apart through a spatial spillover mechanism, such as an import-oriented inflation effect and a trade effect.

In model 7, the indirect effect of tourist satisfaction is more significant than in model 6, and the coefficient is larger. However, the direct and indirect effects of tourist satisfaction are significantly negative in model 8, which means that urban tourist satisfaction negatively influences the urban housing sales price. And, more remarkably, the coefficients of GDP and its spatially lagged term in this model are negative. This may be attributable to the negative effect of GDP, which in fact it is quite common when using econometric models for empirical analysis (Wen et al., 2018[47]; Dai and Wen, 2018[48]; Gong and Lin, 2017[49])

### Robustness tests

#### Impact of urban tourist satisfaction on urban GDP

The paper applies the SDM_dlag to conduct the robustness test.

**Table 9** examines the impact of urban tourist satisfaction on urban GDP income with SDM_dlag, and models 9 and 10 correspond to models 1 and 3, respectively. The empirical results demonstrate that the long-term indirect effects of tourist satisfaction in the two models are positively statistically significant, consistent with the results of models 1 and 3, and the short-term indirect effects are also significantly positive, which demonstrates that not only the long-term but also in the short-term, the urban tourist satisfaction has spatial spillover effect on GDP growth of other cities. This signifies that urban tourists’ satisfaction has a remarkable spatial spillover effect on the GDP growth of other cities in both the long-term and the short-term. However, the long-term direct effect of tourists' satisfaction, which is not statistically significant in models 1 and 3, becomes significant in models 9 and 10, even though the degree of significance is not very high. Moreover, the short-term direct effect of tourists' satisfaction is also negatively significant, so it seems that this result does not meet the expectations, indicating that the influence of tourist satisfaction on the city's GDP income is not so obvious and a city's GDP, especially in the short term, is more likely to be affected by the fixed assets investment, population and technical factors. Indeed, the long-run and short-run direct effects of the fixed assets (represented by “lninvestment”) in the four models are significantly positive.

**Table 9:** SDM_dlag regression results for urban tourist satisfaction and urban GDP.

	Model 9			Model 10	
Lngdp	Coef.	z	lngdp	Coef.	z
**Main**			**Main**		
L1.	0.0698***	3.21	L1.	0.0737***	3.49
lninvestment	0.0470***	4.73	lninvestment	0.0471***	4.73
Lncpi	0.4441	0.72			
Lnsatisfy	-0.1873*	-1.67	lnsatisfy	-0.1948*	-1.73
**Wx**			**Wx**		
Lngdp	0.8134***	9.92	lngdp	0.8149***	9.94
lninvestment	0.0104	0.72	lninvestment	0.0111	0.77
Lncpi	-0.6724	-1.01			
Lnsatisfy	0.2993**	2.22	lnsatisfy	0.2839**	2.18
**SR_Direct**			**SR_Direct**		
lninvestment	0.0468***	4.88	lninvestment	0.0469***	4.88
Lncpi	0.5092	0.86			
Lnsatisfy	-0.1881*	-1.71	lnsatisfy	-0.1832*	-1.71
Lngdp	-0.0011	-0.07	lngdp	-0.0010	-0.07
**SR_Indirect**			**SR_Indirect**		
lninvestment	0.0116	0.76	lninvestment	0.0120	0.82
Lncpi	-0.7282	-1.12			
Lnsatisfy	0.2976**	2.18	lnsatisfy	0.2797**	2.26
Lngdp	0.8139***	18.2	lngdp	0.8140***	17.43
**LR_Direct**			**LR_Direct**		
lninvestment	0.0503***	4.88	lninvestment	0.0506***	4.88
Lncpi	0.5475	0.86			
Lnsatisfy	-0.2022*	-1.71	lnsatisfy	-0.1978*	-1.71
Lngdp	-0.0012	-0.07	lngdp	-0.0012	-0.07
**LR_Indirect**			**LR_Indirect**		
lninvestment	0.0125	0.76	lninvestment	0.0130	0.81
Lncpi	-0.7832	-1.12			
Lnsatisfy	0.3201**	2.18	lnsatisfy	0.3020**	2.26
Lngdp	0.8753***	18.98	lngdp	0.8791***	18.26
**R**^**2**^	0.8127		**R**^**2**^	0.8124	
**Log-likelihood**	616.3		**Log-likelihood**	615.7	

L1. is the first-order lagged dependent variable, and this symbol is the same for the Table 3.7.

Models 10 and 11 in **Table 10** correspond to models 4 and 5, respectively, and the results are basically consistent with model 9, which illustrates that no matter how the cost of living is measured, the long-term indirect effect and the short-term indirect effect of tourists' satisfaction are significantly positive, and urban tourists' satisfaction has a spatial spillover effect on GDP growth of other cities. Nevertheless, the long-term and the short-term direct effects are not either obvious or negative; that is, the mechanism of their economic impact on the city is relatively complex.

**Table 10:** SDM_Lag regression results for urban tourist satisfaction and urban GDP (Continued).

	Model 9			Model 10			Model 11	
lngdp	Coef.	z	lngdp	Coef.	z	lngdp	Coef.	z
**Main**			**Main**			**Main**		
L1.	0.0698***	3.21	L1.	0.0666***	3.01	L1.	0.0696***	3.23
lninvestment	0.0470***	4.73	lninvestment	0.0473***	4.76	lninvestment	0.0472***	4.74
lncpi	0.4441	0.72	lnprice	-0.3405	-1.05	lnhouse	-0.0701	-0.9
lnsatisfy	-0.1873*	-1.67	lnsatisfy	-0.1869*	-1.66	lnsatisfy	-0.2055*	-1.82
**Wx**			**Wx**			**Wx**		
lngdp	0.8134***	9.92	lngdp	0.8071***	9.81	lngdp	0.8112***	9.89
lninvestment	0.0104	0.72	lninvestment	0.0112	0.78	lninvestment	0.0114	0.79
lnsatisfy	0.2993**	2.22	lnsatisfy	0.3275**	2.4	lnsatisfy	0.2564*	1.92
lncpi	-0.6724	-1.01						
**SR_Direct**			**SR_Direct**			**SR_Direct**		
lninvestment	0.0468***	4.88	lninvestment	0.0470***	4.9	lninvestment	0.0469***	4.89
lncpi	0.5092	0.86	lnprice	-0.3049	-0.98	lnhouse	-0.0617	-0.83
lnsatisfy	-0.1881*	-1.71	lnsatisfy	-0.1890*	-1.73	lnsatisfy	-0.2056*	-1.86
lngdp	-0.0011	-0.07	lngdp	-0.0015	-0.1	lngdp	-0.0015	-0.1
**SR_Indirect**			**SR_Indirect**			**SR_Indirect**		
lninvestment	0.0116	0.76	lninvestment	0.0121	0.79	lninvestment	0.0124	0.81
lncpi	-0.7282	-1.12	lnprice	-0.0027	-0.08	lnhouse	-0.0006	-0.08
lnsatisfy	0.2976**	2.18	lnsatisfy	0.3256**	2.33	lnsatisfy	0.2617*	1.92
lngdp	0.8139***	18.2	lngdp	0.8077***	17.84	lngdp	0.8111***	18.17
**LR_Direct**			**LR_Direct**			**LR_Direct**		
lninvestment	0.0503***	4.88	lninvestment	0.0504	4.9	lninvestment	0.0504***	4.89
lncpi	0.5475	0.86	lnprice	-0.3267	-0.98	lnhouse	-0.0663	-0.83
lnsatisfy	-0.2022*	-1.71	lnsatisfy	-0.2025*	-1.73	lnsatisfy	-0.2210*	-1.86
lngdp	-0.0012	-0.07	lngdp	-0.0017	-0.1	lngdp	-0.0017	-0.1
**LR_Indirect**			**LR_Indirect**			**LR_Indirect**		
lninvestment	0.0125	0.76	lninvestment	0.0130	0.79	lninvestment	0.0133	0.8
lncpi	-0.7832	-1.12	lnprice	-0.0032	-0.09	lnhouse	-0.0007	-0.09
lnsatisfy	0.3201**	2.18	lnsatisfy	0.3490**	2.33	lnsatisfy	0.2814*	1.92
lngdp	0.8753***	18.98	lngdp	0.8655***	18.58	lngdp	0.8721***	18.96
**R**^**2**^	0.8127		**R**^**2**^	0.8128	**R**^**2**^	**R**^**2**^	0.8127	
**Log-likelihood**	616.3		**Log-likelihood**	616.5	**Log-likelihood**	616.2		

#### Impact of urban tourist satisfaction on urban cost of living

**Table 11** investigates the impact of urban tourist satisfaction on the urban cost of life with an SDM_dlag; models 12, 13, and 14 correspond to models 6, 7, and 8, respectively. The empirical results signify that, in model 12 and model 13, with CPI and urban commodity consumption price index as dependent variables, respectively, the long-term direct and indirect effects of tourists' satisfaction are not significant, while the long-term direct effects are significant in model 6 and model 7. Moreover, the short-term direct and indirect effects of tourist satisfaction are significantly negative in the SDM_dlag, implying that, in the short term, the enhancement of tourists' satisfaction not only plays a vital role in the reduction of local living costs but also contributes a spatial spillover effect to the living costs of other cities, whereas in the long term, the intra-regional and spatial spillover effects are no longer affected due to the lagged dependent variables.

**Table 11:** SDM_Lag regression results for urban tourist satisfaction and urban cost of living.

	Model 12			Model 13			Model 14	
lncpi	Coef.	z	lnprice	Coef.	z	lnhouse	Coef.	z
**Main**			**Main**			**Main**		
L1.	0.5244***	21.66	L1.	0.6639***	24.96	L1.	0.6552***	31.73
lngdp	0.0005	0.27	lngdp	-0.0002	-0.08	lngdp	-0.0063	-0.68
lninvestment	0.0000	-0.04	lninvestment	0.0005	0.73	lninvestment	-0.0021	-0.83
lnsatisfy	-0.0099*	-1.67	lnsatisfy	-0.0152**	-2.15	lnsatisfy	0.0116	0.41
**Wx**			**Wx**			**Wx**		
lngdp	-0.0037	-1.62	lngdp	0.0012	0.44	lngdp	0.0155	1.44
lninvestment	0.0004	0.6	lninvestment	0.0007	0.81	lninvestment	-0.0016	-0.48
lnsatisfy	-0.0157**	-2.14	lnsatisfy	-0.0274**	-3.11	lnsatisfy	0.0552	1.64
**SR_Direct**			**SR_Direct**			**SR_Direct**		
lngdp	-0.0002	-0.12	lngdp	-0.0001	-0.03	lngdp	-0.0048	-0.57
lninvestment	0.0001	0.25	lninvestment	0.0007	1.17	lninvestment	-0.0022	-0.94
lnsatisfy	-0.0142**	-2.53	lnsatisfy	-0.0214***	-3.19	lnsatisfy	0.0200	0.77
**SR_Indirect**			**SR_Indirect**			**SR_Indirect**		
lngdp	-0.0067**	-2.27	lngdp	0.0022	0.6	lngdp	0.0210*	1.68
lninvestment	0.0009	0.72	lninvestment	0.0017	1.29	lninvestment	-0.0041	-0.87
lnsatisfy	-0.0425***	-3.6	lnsatisfy	-0.0641***	-4.7	lnsatisfy	0.0993**	2.07
**LR_Direct**			**LR_Direct**			**LR_Direct**		
Lngdp	-0.0012	-0.01	lngdp	-0.0026	-0.19	lngdp	-0.0568	-0.11
lninvestment	0.0014	0.07	lninvestment	-0.0028	-0.21	lninvestment	0.0155	0.07
Lnsatisfy	-0.0322	-0.02	lnsatisfy	0.1025	0.26	lnsatisfy	-0.2630	-0.06
**LR_Indirect**			**LR_Indirect**			**LR_Indirect**		
Lngdp	0.0514	0.41	lngdp	-0.0035	-0.24	lngdp	-0.0542	-0.1
lninvestment	-0.0083	-0.35	lninvestment	-0.0046	-0.35	lninvestment	0.0276	0.13
Lnsatisfy	0.4350	0.31	lnsatisfy	0.1628	0.42	lnsatisfy	-0.5256	-0.11
**R**^**2**^	0.6934		**R**^**2**^	0.7602		**R**^**2**^	0.7018	
**Log-likelihood**	2638.4		**Log-likelihood**	2517.6		**Log-likelihood**	1570.6	

When employing model 14, with the housing consumption index as the dependent variable, the direct and indirect effects of tourists' satisfaction, which are negatively significantly in model 8, are no longer significant; the possible reason for this may lie in the addition of the lagged dependent GDP.

## Conclusions and recommendation

### Conclusion of the research

This paper utilizes the Spatial Durbin Model (SDM) to examine the spatial spillover effect of urban tourism satisfaction on urban macroeconomics from a macro perspective, using quarterly data on tourist satisfaction in 35 large and medium-sized cities and major urban macroeconomic variables for 2010 to 2015. This is quite distinct from previous studies that have focused on constructing a tourist satisfaction index and analyzing the factors influencing tourism satisfaction from the micro-level internal composition of tourism. The empirical results show that:

First, when examining the impact of urban tourists' satisfaction on the GDP income of cities using both the SDM and the SDM_with a lagged first-order dependent variable (SDM_dlag), the short-term and long-term indirect effects of log-tourist satisfaction are significantly positive, indicating that the increase in urban tourists’ satisfaction will also have a positive effect on the GDP of other cities. In other words, cities’ satisfaction has a significant positive spatial spillover effect on GDP growth in other cities.

Secondly, as for the influence of urban tourist satisfaction on the cost of urban life in the SDM, taking the consumer price index (CPI), the commodity consumption index, and the housing sales’ price as dependent variables, the long-run direct effect of logarithmic tourist satisfaction is significantly positive, implying that increasing tourist satisfaction will lead to higher prices of consumer goods and housing in the local city; that is, the tourists’ satisfaction has positive intraregional and spatial spillover effects on urban living costs. Meanwhile, the indirect effect is also positively significant, signifying that the enhancement of urban tourists' satisfaction will also have a positive effect on CPI and commodity consumption index in other cities; that is, urban tourist satisfaction has a prominent spatial spillover effect.

Finally, in the SDM_dlag for the regression of urban tourism satisfaction on the cost of urban daily life, the short-term direct and indirect effects of city tourist satisfaction are significantly negative, indicating that tourist satisfaction has intra-regional and spatial spillover effects and that its rise will reduce the cost of living expense in local and other cities in the short-term. However, in the long-term, intra-regional and spatial spillover effects no longer work because of the lagged dependent variables.

### Recommendations

Given the above empirical results, this paper draws the following two recommendations.

On the one hand, urban tourist satisfaction not only affects tourism-related attractions but also influences other macro variables of the national economy, such as urban GDP, a proxy variable for the overall economic level of the city; urban CPI, representing non-durable consumption of urban living cost; and urban housing sales’ price index, a proxy variable for durable consumption in this paper. Therefore, in addition to tourism-related departments, which are responsible for taking measures to facilitate tourist satisfaction, other economic sectors also need to fully grasp the positive long-term spatial spillover effect and short-term negative intra-regional spillover effect and spatial spillover effect of urban tourist satisfaction on not only economic growth but also the cost of living.

On the other hand, in accordance with the macroeconomic import–inflation theory, the price index of a city will affect the price index in its neighboring cities, and, with the remarkable development of Internet economics, it will even influence the price indexes in cities farther away. Consequently, a city's tourist satisfaction will not only affect the price of local necessities and housing construction but will also harm other cities’ cost of living in the long run, reducing the well-being of citizen. For example, in Sanya, a famous tourist city of China that is located in Hainan province, a high degree of tourist satisfaction in the very early days of tourism primarily originated from the relatively low price level, better air quality, and other excellent conditions compared to other cities; however, with the booming numbers of tourists, the local CPI and housing construction prices have soared sharply, which has done considerable damage to the urban tourist satisfaction.
